# Luxation obturatrice de la hanche: un traumatisme rare en pratique sportive

**DOI:** 10.11604/pamj.2015.21.230.4449

**Published:** 2015-07-30

**Authors:** Issam Elouakili, Younes Ouchrif, Redouane Ouakrim, Omar Lamrani, Mohammed Kharmaz, Farid Ismael, Abdou Lahlou, Ahmed El Bardouni, Mustapha Mahfoud, Mohamed Saleh Berrada, Moradh El Yaacoubi

**Affiliations:** 1 Service de Chirurgie Orthopédique, CHU de Rabat, Maroc

**Keywords:** Luxation, obturatrice, complication, roller, dislocation, obturator, complication, roller

## Abstract

Les luxations antérieures traumatiques de la hanche sans fracture du cotyle ou de la tête fémorale sont rares. Elles sont souvent secondaires à des accidents de haute énergie cinétique. La prise en charge thérapeutique nécessite un chirurgien vigilant et prévenu du risque de complications. Nous rapportons le cas d'une luxation obturatrice (antéro-inférieure) chez un jeune de 18 ans pratiquant le roller

## Introduction

Les luxations antérieures traumatiques de la hanche sans fracture du cotyle ou de la têtefémorale sont rares. Elles sont souvent secondaires à des accidents de la voie publique et rarement rencontrées dans la pratique sportive. Nous rapportons le cas d'une luxation obturatrice (antéro-inférieure) chez un jeune de 18 ans pratiquant le roller.

## Patient et observation

**Figure 1:**
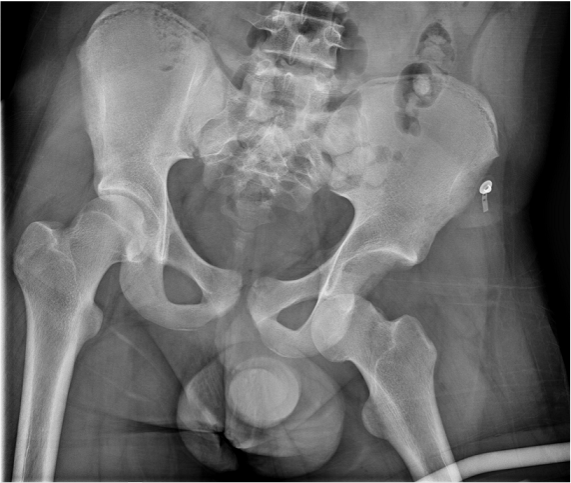
Radiographie montrant la luxation antéro-inferieur gauche

**Figure 2:**
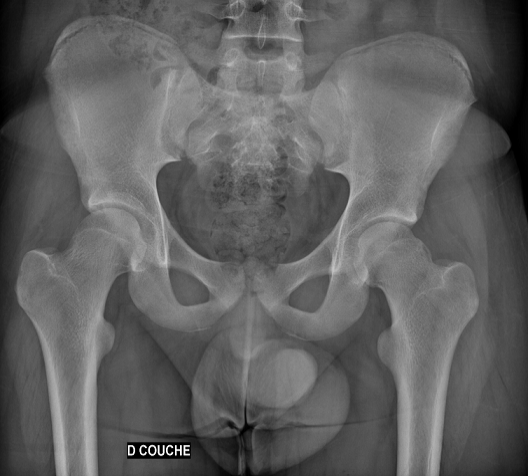
Radiographie bassin de face montrant la réduction de la luxation de la hanche gauche

**Figure 3:**
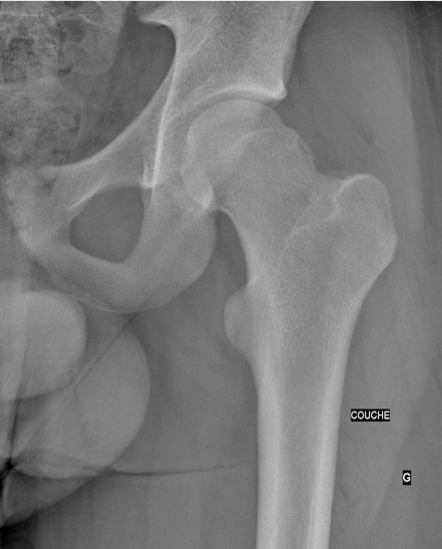
Radiographie de la hanche gauche montrant la réduction de la luxation

**Figure 4:**
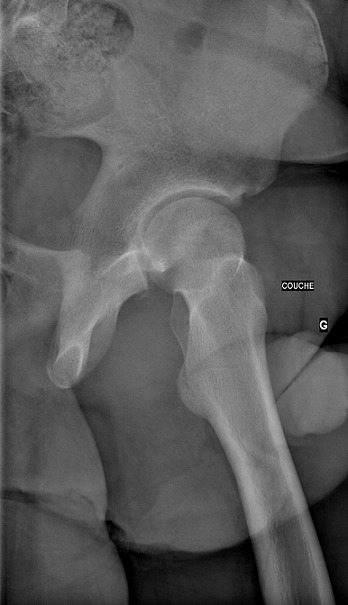
Radiographie de profile montrant la réduction de la luxation de la hanche gauche

**Figure 5:**
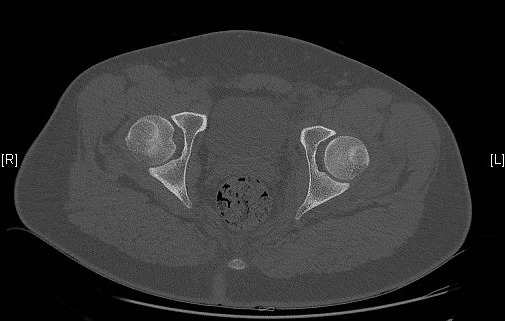
Scanner après réduction n'objective aucune lésion associée ou corps étranger

**Figure 6:**
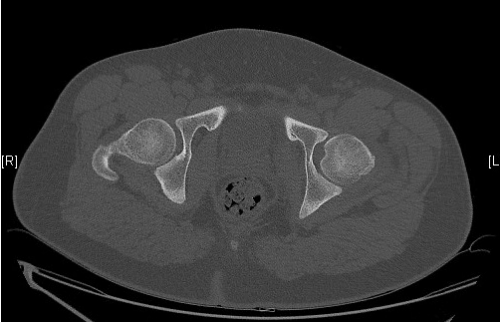
Scanner avec coupe plus distale n'objective pas de lésion associée ou corps étranger

Il s'agit d'un patient de 18 ans pratiquant le roller depuis 4 ans. Qui a subi un traumatisme lors de la pratique sportive en touchant la roue arrière du vélo de son collègue. Cela a occasionné chez lui un traumatisme de la hanche gauche. L'examen initial a retrouvé une impotence fonctionnelle totale du membre inférieur gauche, avec une hanche en flexion abduction rotation externe. L'examen vasculo-nerveux était sans anomalie ainsi que l'examen cutané. Une radiographie pratique en urgence (Figure 1) a montré une luxation obturatrice de la hanche gauche. Uneréduction en urgence sous anesthésie générale et curarisation a été réalisée au bloc opératoire en utilisant la manoeuvre suivante: Une traction initiale dans l'axe du membre suivie d'une flexion de la hanche en rotation interne et en abduction tout en gardant la traction. La radiographie de contrôle était satisfaisante (Figure 2, Figure 3, Figure 4) et une TDM post-réductionnelle(Figure 5, Figure 6) a été pratiquée et qui a confirmé la réduction ainsi que l'absence de lésion associée et de fragment intra articulaire. Une décharge de 15 jours a été prescrite tout en évitant les mouvements en rotation externe pour une durée de 4 semaines. La reprise de l'activité sportive était sans problème au 6ème mois.

## Discussion

Les luxations traumatiques sont rarement isolées. Dans la plupart des cas, elles sont associées à des fractures du cotyle ou de la tête fémorale. La luxation obturatrice représente 6 à 10% de luxations rapportées dans la littérature [1]. Le mécanisme de survenue de cette entité est un mouvement en flexion, Abduction et rotation externe forcée [2, 3]. C'est ce mécanisme qui explique la luxation dans notre cas: L'impaction s'est faite à forte vitesse sur la face interne du pied entrainant une rotation externe forcée brutale de la hanche, celle-ci étant en flexion. Catonné et al [4] ont rapporté 2 cas similaires dans un accident de ski nautique. Les lésions capsulaires antérieures sont constantes [5]. Ces lésions peuvent entrainer une irréductibilité par effet boutonnière [6]. Egalement les lésions ostéo-articulaires sont très fréquentes et dépendent du mécanisme et de la violence du traumatisme initial. Les fractures de la tête fémorale surviendraient dans plus de 50% des luxations antérieures [4]. Les lésions cartilagineuses se rencontrent dans 63% des cas selon les séries rapportées dans la littérature [4]. Certains auteurs pratiquent des arthroscopies systématiques après réduction, vu la fréquence importante de corps étranger intra articulaire et qui peuvent passer inaperçu sur le scanner [5]. Des hématomes peuvent se rencontrer en cas de lésion vasculaire voire même une ostéonécrose de la tête fémorale en cas d'atteinte des vaisseaux circonflexes.

Le traitement des luxations obturatrices isolées est orthopédique. Cette réduction peut être difficile chez les sujets musclés. Il est recommandé de la pratiquer sous anesthésie générale avec curarisation et il est capital de ne pas entrainer de fracture du col lors des manoeuvres de réduction [5]. Les modalités de réduction sont très discutées dans la littérature. Epstein [2] et Brav [7] recommandent une traction dans l'axe du fémur suivie d'une flexion progressive de la hanche en rotation interne et en abduction, tout en maintenant la traction. Toms et al [8] ont critiqué l'abduction dans la manoeuvre de réduction vu que la hanche est déjà en abduction forcée. Ils ont condamné également le mouvement en rotation interne forcée qui expliquerait la fracture du col fémorale décrit par certains [4, 9]. Ils préconisent d'utiliser la table orthopédique et d'associer à la traction axiale, une traction latérale de la cuisse puis de relâcher progressivement la traction tout en imprégnant un mouvement d'adduction rotation interne. Ces discussions attirent l'attention sur les difficultés de réduction et le risque important de complications qui peuvent emmener à un abord chirurgical pour une réduction sanglante. Les suites après réduction orthopédique ou chirurgicale ne sont pas consensuelles. Actuellement il n'y a aucun argument scientifique qui justifie l'intérêt de la traction et de la décharge dans la diminution du risque de nécrose céphalique de la tête fémorale [4] Catonné et al recommandent un appui précoce soulagé puis total à j15 avec éviction de la rotation externe pendant 3 semaines dans le cadre des luxations antérieures [4]. Le risque de survenu de nécrose céphalique augmente avec le retard de réduction. Ce risque et de 30% chez l'adulte [10]. Hoogard [11] a observé 47% de nécrose quand le délai de réduction dépassait les 6h. Mais ces chiffres concernent en majorité des lésions associées à des fractures du cotyle ou de la tête fémorale, ce taux est certainement inférieur dans les luxations isolées.

## Conclusion

La luxation obturatrice sans fracture n'est pas fréquente en pratique sportive. Sa réduction n'est pas toujours facile et peut changer complètement le pronostic thérapeutique.
